# Barriers and facilitators to implementing a new regulation restricting antimicrobial use in dairy production in Québec, Canada: A qualitative study

**DOI:** 10.3389/fvets.2023.1025781

**Published:** 2023-03-16

**Authors:** Nikky Millar, Simon Dufour, Hélène Lardé, Jean-Philippe Roy, Catherine Belloc, David Francoz, Marie-Ève Paradis, Marie Archambault, John Morris Fairbrother, Cécile Aenishaenslin

**Affiliations:** ^1^Department of Pathology and Microbiology, Faculty of Veterinary Medicine, Université de Montréal, Saint-Hyacinthe, QC, Canada; ^2^Groupe de recherche en épidémiologie des zoonoses et santé publique (GREZOSP), Faculty of Veterinary Medicine, Université de Montréal, Saint-Hyacinthe, QC, Canada; ^3^Centre de recherche en santé publique, Université de Montréal et Centre intégré de santé et de services sociaux du Québec du Centre-Sud-de-l'Île-de-Montréal, Montréal, QC, Canada; ^4^Fond de recherche Nature et technologies du Québec (FRQNT)—Regroupement FRQNT Op+lait, Saint-Hyacinthe, QC, Canada; ^5^Ross University School of Veterinary Medicine, Basseterre, Saint Kitts and Nevis; ^6^Department of Clinical Sciences, Faculty of Veterinary Medicine, Université de Montréal, Saint-Hyacinthe, QC, Canada; ^7^Oniris, INRAE, BIOEPAR, Nantes, France; ^8^Association des médecins vétérinaires praticiens du Québec, Saint-Hyacinthe, QC, Canada

**Keywords:** antimicrobials, antimicrobial resistance, legislation, animals, behavior, individual interview, qualitative research

## Abstract

With the emergence of antimicrobial resistance (AMR), many countries are implementing restrictive regulations to reduce antimicrobial use (AMU) in animal production. Although these measures are effective at the national level, their implementation may generate challenges for producers and veterinarians. The objective of this study was to explore the barriers and facilitators of implementing a new regulation restricting the use of antimicrobials of very high importance for human health in the dairy production sector in the province of Québec, Canada. Individual interviews were conducted with fifteen veterinarians and twenty-seven dairy producers. Thematic analysis was performed based on the COM-B model of behavior change (capability-opportunity-motivation-behavior). Our results indicated that the lack of availability of alternative treatments, the long delays related to diagnostic tests and the fear of economic consequences were major barriers to the implementation of the regulation. A small number of producers also perceived that the regulation negatively impacted the health and wellbeing of their animals. Additionally, participants acknowledged the importance of early education and training to better understand the purpose of the regulation and increase its acceptability. Lastly, most participants reported that they had not only reduced their use of antimicrobials of very high importance for human health following the regulation, but they had also increased preventive practices on their farm. This study reveals that the implementation of restrictive regulations to reduce AMU in animal production can lead to multiple challenges in practice. Our results highlight the need for better communication and training of producers and veterinarians before and during the implementation of similar regulations in the future and underline the importance of measuring the direct and indirect impacts of those regulations on productivity and on animal health and wellbeing.

## 1. Introduction

In the last few decades, the use of antimicrobials (AMs) in humans and animals have contributed to the emergence of antimicrobial resistance (AMR), which impacts the health of animals and humans by reducing therapeutic options. The extent to which antimicrobial use (AMU) in food-producing animals contributes to the burden of AMR in humans is still unknown. However, growing evidence supports an association between AMR circulating in animals and in humans, especially in the context of close human-animal contacts (1). In order to tackle AMR, a global action plan was developed in 2015 by the World Health Organization (WHO) in collaboration with the Food and Agriculture Organization of the United Nations (FAO) and the World Organization for Animal Health (WOAH) (2). The global action plan has five objectives which underscore the need for harmonized, global and immediate actions against AMR: improving awareness and understanding of AMR, strengthening surveillance and research, reducing the incidence of infection, optimizing the use of antimicrobial medicines, and ensuring sustainable investment in mitigating AMR (2).

Several countries have already implemented measures to monitor and reduce AMU in animals, including the implementation of new regulations to restrict AMU in animal production. In the province of Québec, Canada, a new regulation restricting the use of AMs of very high importance for human medicine in all food-producing animals was implemented in February 2019 (3). These AMs are known as Category 1 AMs in Canada and mainly refer to third- and fourth-generation cephalosporins, polymyxins, and fluoroquinolones in animal production. The new regulation prohibits the use of these AMs for preventive (the treatment of healthy animals at risk of infection) purposes in food-producing animals. It also specifies that veterinarians must justify that an AM of lesser importance for humans would not be effective or available before prescribing a Category 1 AM for therapeutic (the treatment of sick animals) or metaphylactic (the treatment of both diseased and healthy animals which are grouped together due to their close contact) purposes in food-producing animals. Category 1 AMs are mainly used to treat mastitis and respiratory diseases in dairy farms (4). Before the implementation of this regulation, 15% of AMs used in Québec's dairies were Category 1 AMs, mainly injectable and intramammary formulations labeled for treating clinical mastitis during lactation, and dry-off intramammary formulations (4). Moreover, a recent study in the province of Québec, demonstrated that the prevalence of AMR was low for Category 1 AMs, but there was a high prevalence of ESBL/AmpC-producing *Escherichia Coli* (*E. Coli*) in Québec dairies (5). Following the implementation of this regulation in dairy farms, a significant decrease in Category 1 AMU of 19 DCDbovCA/herd-year (95% CI [14.8, 24.2]) was observed, as well as a decrease in the prevalence of multidrug resistance of *E. Coli* isolates from fecal and manure pit samples in dairy farms from 83 to 71% (*p* = 0.05) (6, 7).

Although effective, this type of regulation involves significant changes in practices for producers and veterinarians. A study conducted in Canada reported that producers feared a decrease in animals' welfare and an increase in production costs if they had to reduce their AMU (8). Other studies conducted in Europe and New Zealand showed that, although producers generally acknowledge the importance of reducing AMU to minimize their impact on AMR, it was not always possible for them to significantly reduce their use because of animal health and economic factors (9–11). A better understanding of the barriers and facilitators related to these behavior changes is needed in order to prevent and mitigate collateral impacts of such regulations.

This study had two objectives. The first objective was to investigate the barriers and facilitators associated with AMU-related behavioral changes in dairy producers and veterinarians after the implementation of a regulation restricting the use of Category 1 AMs in Québec, Canada. The second objective was to investigate the perceived collateral impacts (both positive and negative impacts that were not expected) of this regulation 2 years after its implementation.

## 2. Material and methods

### 2.1. Positionally and reflexibility statement

All researchers included in this study are veterinarians and two of them have advanced degrees in epidemiology and qualitative research. Five of the authors have worked as veterinarians on farms (J-PR, M-EP, and SD) or in a large animal hospital setting (HL and DF). The first author (NM) performed the interviews, the data analysis and manuscript writing. N.M. is a woman, a doctoral student in epidemiology, focusing her work on AMR and AMU in the animal production industry. As a veterinarian, N.M. understands the role of AMU with respect to AMR and understands the complexity of decisions regarding AMU on farms. She has not worked on farms as a veterinarian. N.M. did not make prior acquaintance with the participants before the interviews.

### 2.2. Study design and theoretical framework

A qualitative study using individual semi-structured interviews with dairy producers and veterinarians was conducted. The study aimed to investigate how different individuals and contextual factors influenced changes in AMU practices in dairy farms, based on the COM-B model (12). The COM-B model is a theoretical framework which has been developed to characterize, design and analyze behavior change interventions. It postulates that people's behavior is influenced by three different types of drivers (which can be separated into two components): capabilities (physical or psychological), opportunities (social or physical), and motivations (automatic or reflective). In the context of this study, physical capability was defined as the physical skills and abilities of producers and veterinarians that are required to change AMU behavior. Psychological capability referred to whether producers and veterinarians had sufficient knowledge or the right attitudes to change their AMU practices. Physical opportunity was defined as external elements that facilitate or impede behavior changes, such as time and resources (i.e., human, financial). Social opportunity referred to social factors that could help producers and veterinarians change AMU behavior (i.e., influence of a peer). Reflective motivation was defined as any reflective process that supports producers and veterinarians' decision-making regarding the regulation. Automatic motivation refers to any unintentional feelings that affect producers and veterinarians' decision-making. Table 1 shows the different types of questions that were explored for each of the drivers and their components. The study protocol was reviewed and approved by the Université de Montréal's ethical board (Comité d'éthique de la recherche en sciences et en santé; certificate number CERSES-20-158-D).

**Table 1:** Components of the COM-B model and related elements investigated in the interviews of 27 dairy producers and 15 dairy cattle veterinarians about barriers and facilitators associated with the implementation of a regulation restricting the use of very high importance antimicrobials for human medicine in animal production in the province of Québec, Canada.

**COM-B components**	**What needs to happen for the target behavior to occur?^a^ Examples of questions**
Physical capability^b^	To what extent producers/veterinarians can use alternatives to Category 1 antimicrobials in terms of the duration of treatments and the number of injections per day? (i.e., alternative antimicrobials to Category 1 antimicrobials required more injections per day. Sometimes, producers don't have the time to catch the cow, tie it up, and give the injections 2–3 times per day.)
	Is it more difficult to administer proper treatment to the cows, because alternative treatments are longer? (i.e., alternative antimicrobials required more injections during more days. Therefore, some animals could become reluctant to the injections it could, then, become difficult for the producer to give the injection.)
Psychological capability^c^	How well do producers/veterinarians know and understand the regulation?
	To what extent producers/veterinarians know about alternatives to Category 1 antimicrobials or diagnostic tests?
Physical opportunity^d^	How easy is it for producers/veterinarians to have access to and use alternative antimicrobials and diagnostic tests?
	How alternatives to Category 1 antimicrobials allow for sufficient care of animals?
Social opportunity^e^	How are peers implementing required changes?
	e.g., what is the influence of relatives and other peers on producers/veterinarians' behavior? To what extent do veterinarians have an impact on behavior changes on farms?
Reflective motivation^f^	How do producers/veterinarians perceived the regulation before its implementation?
	How do producers/veterinarians perceive the consequences of using Category 1 antimicrobials and the impact of antimicrobial resistance globally?
	What are producers/veterinarians' personal objectives for antimicrobial use reduction?
Automatic motivation^g^	How do producers/veterinarians feel about the regulation and changing their habits regarding Category 1 antimicrobial use?
	How do producers/veterinarians feel about the reduction of antimicrobial use in animal production in general?

^a^The target behavior is the reduction in Category 1 antimicrobial use.

^b^Definition: Having the appropriate physical skills, physical strength to engage a specific behavior.

^c^Definition: Having the appropriate knowledge and psychological strength to engage a specific behavior.

^d^Definition: Environment (time, localization) that provide adequate opportunities to make a behavior possible.

^e^Definition: Social factors (cultural norms, social cues) that provides opportunities to make a behavior possible.

^f^Definition: Reflective process which influence decision-making and, therefore, our behavior.

^g^Definition: Automatic process (desires, impulses, inhibitions) which influence decision-making and, therefore, our behavior.

### 2.3. Sampling and recruitment

Two approaches were used successively to recruit producers and veterinarians for this study. First, a list of 87 producers who had already agreed to participate in a larger project about AMU and AMR was used to recruit twelve producers (6). Four producers from that list for each of the three regions targeted by this larger project (Estrie, Montérégie and Center-Du-Québec; province of Québec, Canada) were randomly invited to participate. A second recruitment phase was conducted to recruit producers and veterinarians from all regions of the province. Invitations to participate were sent by email to all dairy producers and veterinarians of the province of Québec working with dairies *via* the milk producer's association [Les Producteurs de lait du Québec (PLQ)] and the veterinary association [Association des médecins vétérinaires praticiens du Québec (AMVPQ)]. Interested producers and veterinarians were invited to complete a short questionnaire to assess inclusion criteria and were then contacted by phone for further explanation of the project and to schedule a phone interview. The only inclusion criteria for the study was that participants (dairy producers and veterinarians) had been working with dairy cows in the province of Québec since at least 2018 (one year before the implementation of the regulation). Participant's written consent was obtained before the interview.

### 2.4. Data collection

An interview guide was developed by the research team based mainly on the COM-B model (Supplementary material 1). It consisted of five open-ended questions investigating the different factors described previously, including knowledge about AMU and AMR and the regulation, attitudes toward the regulation, barriers and elements that facilitated changes in their practices, and perceived collateral impacts of the new regulation. Socio-demographic information was also collected to enable comparative analysis between subgroups. For producers, it consisted of the number of years of experience in dairy production, the number of lactating cows on their farms, cattle-housing methods (tie-stall barn vs. free-housing system), the region of their farm and gender of the participant. Veterinarians were required to indicate their number of years in practice, their gender, their type of practice, and the region of practice. To ensure the clarity, completeness and comprehensiveness of the interview guide, interviews were pre-tested with two dairy producers and one veterinarian who were not contributing to the official interviews. Then, interviews were conducted by phone between January and April 2021 by N.M. These were intended to last about 35 min and were recorded. Interviews were performed in French.

### 2.5. Data analysis

All interviews were transcribed *ad verbatim* and all elements that would allow the identification of the participants were removed at this stage. Thematic analysis was performed using both a deductive and an inductive approach. A codebook was developed in two steps (Supplementary material 2). First, the COM-B model was used to define major categories of themes (capability, opportunity, and motivation) adapted to the context of the study. Second, transcripts were read by N.M., and new codes were created and defined based on emerging themes using a deductive approach. These emerging themes were then classified as capability-opportunity-motivation or as collateral impacts. Codes' definitions were discussed until an agreement was reached between researchers (NM, CA). N.M. then coded all transcripts using the software NVivo 12.0. The codebook was refined during the process, when necessary, in consultation with C.A. The barriers and facilitators that emerged from the analysis were grouped into three categories based on the COM-B model drivers: capability, opportunity, motivation. Each of these categories was divided into two components as described above. Interviews were translated into English for the manuscript.

## 3. Results

Twenty-seven producers agreed to participate in the study (12 from a previous larger project and 15 from the e-mail invitation to all producers). A total of 17 veterinarians responded to the e-mail invitation that was sent to all veterinarians who are members of the AMVPQ and 15 of them agreed to participate in the study. The recruitment ended for producers and veterinarians when saturation point was reached for all the topics; i.e., there was no new emerging themes during the last individual interviews. The interviews lasted 19 to 40 min with a mean of 24 min. Socio-demographic characteristics of the participants are shown in Table 2.

**Table 2:** Socio-demographic characteristics of the 27 dairy producers and 15 veterinarians taking part in an individual interview about barriers and facilitators to the implementation of a regulation restricting the use of very high importance antimicrobials for human medicine in animal production in the province of Québec, Canada.

	**Producers**		**Veterinarians**	
	***n*** **/27**	**%**	***n*** **/15**	**%**
**Gender**				
Women	5	18.5	5	33.3
Men	22	81.5	10	66.6
**Region**				
Montérégie	4	14.8	3	20.0
Laurentides-Lanaudière-Laval	1	3.7	4	26.7
Chaudière-Appalaches	1	3.7	2	13.3
Bas-Saint-Laurent	1	3.7	2	13.3
Centre-Du-Québec	9	33.3	2	13.3
Estrie	7	25.9	1	6.7
Saguenay-Lac-Saint-Jean	1	3.7	1	6.7
Abitibi-Témiscamingue	1	3.7	—	—
Mauricie	1	3.7	—	—
Capitale-Nationale	1	3.7	—	—
**Experience**				
<20 years	14	51.9	13	86.7
20 years and more	13	48.1	2	13.3
**Herd size**				
<50 lactating cows (26%)	7	26.0	—	—
51–70 lactating cows (30%)	9	33.3	—	—
71–100 lactating cows (30%)	9	33.3	—	—
100 lactating cows (7%)	2	7.4	—	—
**Milking system**				
Tie stall	15	55.6	—	—
Free stall	12	44.4	—	—

### 3.1. Barriers and facilitators to the regulation implementation

**Figure 1:**
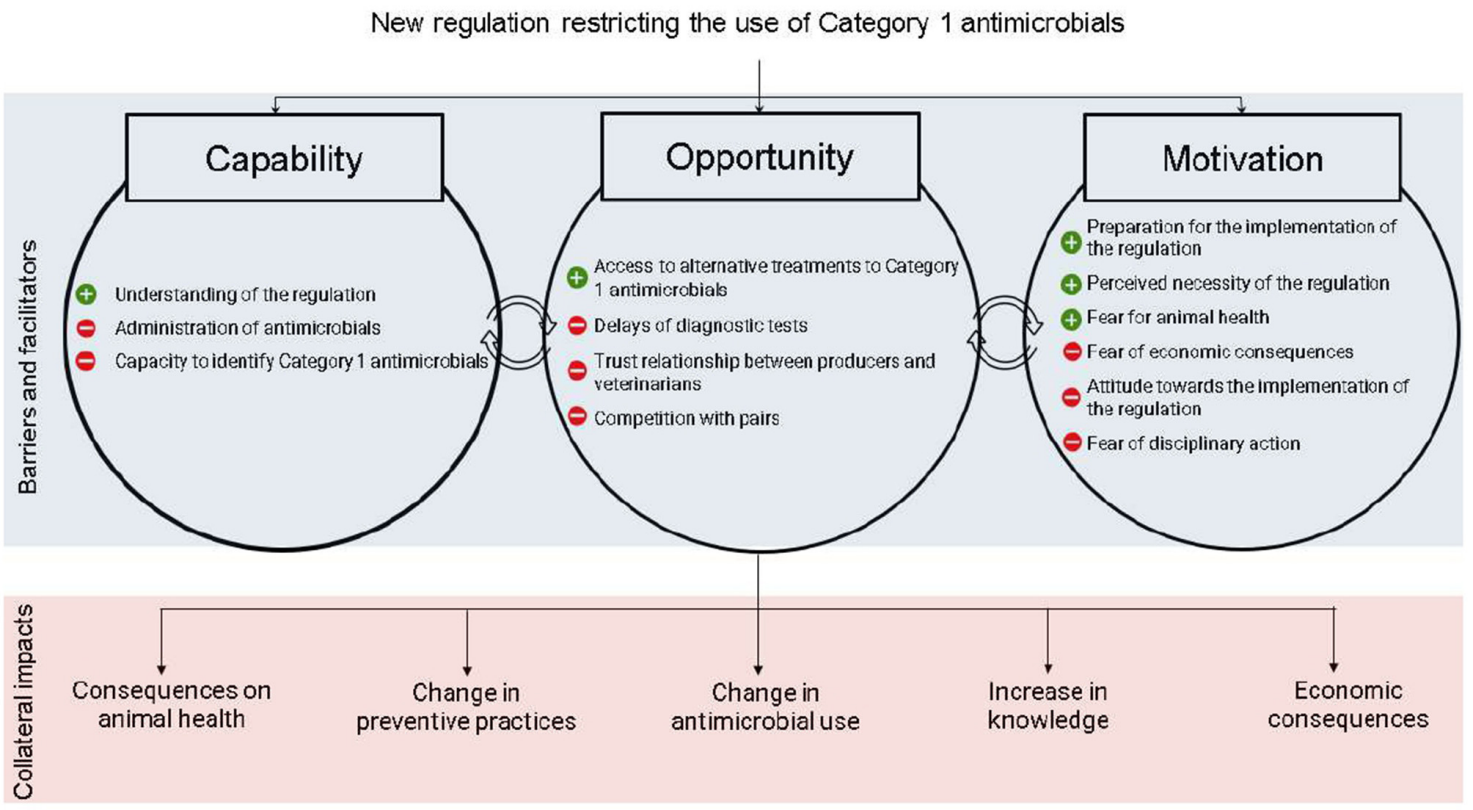
Barriers, facilitators, and perceived collateral impacts of 15 veterinarians and 27 dairy producers who took part in an individual interview about the implementation of regulation restricting the use of very high important antimicrobials in the province of Québec, Canada.

Physical capability, social opportunities, automatic motivation and reflective motivation were found to be minor categories of barriers to the implementation of the regulation, whereas psychological capability and physical opportunity were found to be major types of barriers. Figure 1 shows the themes that emerged for each of the drivers.

#### 3.1.1. Physical capability

One theme emerged in relation to physical capability and was related to the difficulties associated with the administration of alternative treatments to Category 1 AMs. However, this was not found to be an important barrier for most producers and veterinarians.

Indeed, six producers reported that it is now difficult to treat cows because alternatives to Category 1 AMs require multiple injections as opposed to the latter. One producer mentioned: “*when it has been 5 or 6 days that you [the producer] treat your cow, she [the cow] doesn't want to see you anymore and neither do you. I'm getting older and, at some point, you must put on halters and tie up the legs and everything to treat her*.” (Producer 1). Another mentioned: “*Normally, if I put [ceftiofur] on, I only give her an injection [the cow] once. It heals well. Today, I have a cow, I have to give her an anti-inflammatory […]. After that, I have to give her four or five days of [spectinomycin]. It's two shots, let's say, morning and night during 3, 4 days, so it's an inconvenience. It causes more stress for the cow*.” (Producer 14).

#### 3.1.2. Psychological capability

Important factors related to the psychological capability included the producers' understanding of the regulation and their capacity to identify Category 1 AMs. Generally, veterinarians reported that producers had a good understanding of AMs' categorization. However, two producers did not understand why some AMs were categorized as Category 1 AMs: “*The fears I [the producer] had were that there were drugs that I didn't understand why they were in that class [Category 1 AMs] of drugs*.” (Producer 15*)*.

On the other hand, there was a lot of heterogeneity about producers' and veterinarians' knowledge regarding the regulation. Many veterinarians felt that parts of the regulatory text were unclear, leaving room for interpretation. Consequently, they felt that it resulted in an inconsistent application of the regulation. One veterinarian mentioned: “*I think that the law is not applied in the same way everywhere. Is it because of a lack of alternatives or […] a lack of understanding of the regulation? […] The law is not very clear. In some situations, I don't know what we can do or can't do. For example, when you don't have an alternative to Category 1. We don't know if we can use it or not*.” (Veterinarian 14). In general, producers had similar perspectives about their understanding of the regulation: “*The regulation doesn't seem to be enforced in the same way by all clinics*.” (Producer 27).

#### 3.1.3. Physical opportunity

Two main themes emerged in relation to physical opportunity and were related to the difficulty of accessing alternative treatments to Category 1 AMs and the delays associated with the importance of performing diagnostic tests prior to treatments. These two themes were found to be important barriers to the implementation of the regulation.

Many veterinarians reported that they faced difficulties to change their behavior regarding AMU practices because alternative treatments to Category 1 AMs were not available after the implementation of the regulation due to shortages of several alternative AMs: “*The fact that for the last year or so, there have been many, many back-order products, has changed the situation very little with regard to Category 1 products*.” (Veterinarian 1). Producers' perceptions regarding availability of Category 1 alternatives were less homogeneous, although a small number of producers also explained that they had problems with the availability of alternative to Category 1 AMs.

All producers and several veterinarians explained that they had to wait several days after sampling before receiving test results. One veterinarian explained: “*The problem is that it takes a long time to get an answer [of the results of a diagnostic test] and you can't wait that long to treat acute mastitis*.” (Veterinarian 14). Producers also reported problems accessing diagnostic tests, especially for those living in regions where access to veterinary services is challenging: “*It doesn't work [performing diagnostic tests], and the clinic is far away. The clinic is an hour and a half away from our house. So, if I take a milk sample, I don't even have a veterinarian that comes to my area every day. So, I must go and take it for an hour and a half away and I have to come back. Anyway, it's crap, so no, we gave up on that*.” (Producer 1).

#### 3.1.4. Social opportunity

Main themes related to social opportunity were the trust relationship between producers and veterinarians, which was a facilitator for producers and veterinarians, and competition with peers, which was, on the other hand, a barrier for producers and veterinarians.

All producers valued veterinarians' recommendations about the regulation, AMU and preventive practices. One producer explained: “*Again, I [producer] have no problem stopping for half an hour to chat with my veterinarian, exchange information and pay for it. […] I am happy to do it, I don't mind paying for it. I find that I probably have better veterinary services*.” (Producer 2). This trust relationship seemed to have increased acceptability and motivations about the regulation and facilitated changes in practices for many producers.

On the other hand, producers argued that the new regulation did not give them the opportunity to compete with the other provinces. They seem to perceive that they were disadvantaged compared to other provinces: “*What is aberrant is that the milk from New Brunswick and Ontario flows into Québec and in New Brunswick and Ontario, they do not have the same laws as here*.” (Producer 1). *One* veterinarian also explained: “*There are many producers who feel that it is unfair that in Québec, Category 1 AMs are banned, but not in the rest of Canada. It [the change in regulation] did not help [the producers] change their perception*.” (Veterinarian 14). This perception clearly negatively impacted motivation for some producers to implement changes.

#### 3.1.5. Reflective motivation

Four important themes emerged with regard to reflective motivation: the preparation for the implementation of the regulation, the perceived necessity for the regulation, fear for animal health and welfare and fear of negative economic consequences. These acted as barriers to some producers and veterinarians and as facilitators for others.

Most veterinarians reported that they learnt about the regulation several months in advance during mandatory continuous professional training. However, seven veterinarians reported that they did not want to inform the producers before the officialization of the regulation which was only 6 months before its implementation: “*I think that, as professionals, we knew it early enough, but for the producers, they seemed to think that it was presented to them too late. That's probably because we had been informed a long time ago, but before it was official, it had probably not been disclosed to the clientele*” (Veterinarian 5). However, perceptions of producers about the delay between the announcement and implementation were heterogenous. Seven producers reported having heard about the regulation several months in advance and perceived this delay was appropriate: “*I still had time to make changes or to really prepare for the change*” (Producer 2*). Six* producers had opposite perceptions and were unable to change their practices adequately: “*I found out at the last minute. Which made me angry too, because I didn't have time to prepare*.” (Producer 27).

All veterinarians thought that the regulation was necessary to reduce the use of Category 1 AMs and to protect public health. For them, it seemed necessary that producers understand the concept of AMR: “*Of course, there are always reluctant people who continue to complain, but once we explain this regulation to them and once we make them realize that it can be dangerous to misuse Category 1 AMs for them and for their children, and when we show them the statistics of resistance to AMs, when we talk about real cases that have come out in the newspapers or things like that, let's admit it, it makes them think more and they understand the impact and the importance of using them judiciously*.” (Veterinarian 12). However, producers' perceptions were less homogeneous. Some of them agreed on the necessity of the regulation, but others felt that it targets the wrong sector. According to two producers, the quantity of AMs that they used didn't impact AMR in humans: “*Resistance does not come from animals; it comes from humans. So, you're not knocking on the right door*.” (Producer 25). Other producers explained: “*I think it's a good thing, we have to be careful about the antibiotics we use in order to protect the health of humans*.” (Producer 10).

While veterinarians did not mention anything about animal health and welfare, a small number of producers mentioned that they believe the regulation could negatively impact animal health and welfare. Those producers were less motivated to change their practices regarding AMU: “*We have gone backwards. We have moved forward, I can understand, for public health, sure maybe we have, but for animals, we are moving backwards*.” (Producer 19).

While veterinarians did not fear any negative economic consequences for their profession, producers had the opposite perception on this topic, as all producers participating in the study feared negative economic impacts for their industry following the implementation of the regulation. Producers reported that diagnostic tests were expensive for them, and that milk withdrawal time associated with the use of alternative AMs increased the amount of discarded milk and thus, a loss of benefits: “*For sure it brings additional tests, therefore additional expenses*” (Producer 24). One veterinarian also shared: “*They* (producers) *find it expensive to test milk. Also, with other categories of AMs, there is a withdrawal time in the milk [The only AM available without milk withdrawal time in dairies in Canada is a Category 1 AM, a 3rd generation cephalosporin]. So, many producers find that they are going to lose money*.” (Veterinarian 15).

#### 3.1.6. Automatic motivation

Two main themes emerged in relation to automatic motivation: attitudes toward the regulation and fear of disciplinary sanctions. Attitudes acted both as a barrier for some producers and veterinarians and a facilitator for others. The second theme (fear of disciplinary sanctions) was a facilitator for most participants.

The attitude of veterinarians toward the regulation was mostly positive: “*I [the veterinarian] was optimistic. I think it was a good thing. We were abusing AMs*.” (Veterinarian 14). Only two producers were confident and explained that they were willing to change their behavior if they were trusted to make decisions and they were given flexibility. One producer explained: “*I [the producer] will adapt to all regulations as long as we are given some latitude and some judgment, on the farm, in our practices*.” (Producer 24). On the other hand, most producers and two veterinarians had a negative attitude toward the regulation. One producer mentioned: “*We [the producers] were really surprised and shocked because, of course, it was part of our routine. [...]*” (Producer 1).

A small number of veterinarians expressed their concerns about disciplinary actions if they prescribed Category 1 AMs: “*We're really limited in terms of tools right now, and we've always in the back of our minds that we're going to be inspected at some point..*.” (Veterinarian 3). Three producers also expressed that they feared being penalized for using Category 1 AMs: “*We might be able to get Category 1 AMs without prescription from a neighbor, but if you use it, it's going to take a prescription to confirm that you had the right to use it. I'm not any further ahead. I'm going to get penalize by Public Health services. I don't have the choice to follow the regulation […]* “(Producer 19).

### 3.2. Perceived impacts of the implementation of the regulation

Five main themes emerged in relation to the perceived collateral impacts of the regulation: the perceived changes in AMU, an increase in knowledge and awareness, perceived negative economic consequences, perceived negative consequences for animal health and welfare and perceived increase in preventive activities.

Veterinarians' perception of AMU after the implementation of the regulation were unanimous. They perceived that there was a considerable improvement in AMU practices. They reported that they prescribed fewer Category 1 AMs, and they perceived that, in general, producers were more aware of the consequences of AMU: “*Drugs like [ceftiofur] for respiratory problems that were given by injection, I used to sell a lot of them regularly and now, I sold maybe six vials in the last year*.” (Veterinarian 13). Producers' perceptions were less homogeneous. Some producers perceived that the regulation changed their AMU practices for the best. One producer mentioned: “*I had [before the implementation of the regulation] protocols that when a cow didn't deliver, I would inject her with [ceftiofur] right away. It was like a preventive measure. It wasn't necessarily such a good way to do it, but it was to prevent problems from occurring. So, since then, I don't do it anymore, but I hadn't had any problems either, so it's a good thing not to do it either*.” (Producer 21). On the other hand, difficulties to administrate alternative AMs could lead some producers not to follow the indications of the prescriptions, as one of the producers explained: “*Even if I am supposed to inject them twice a day, I don't do it. I give them an injection just once because at twice a day, there are cows that don't want to see you anymore*.” (Producer 1).

Veterinarians perceived that the implementation of the regulation didn't have any impact on animal health and welfare: “*In our clinic, we did a small study internally with the data we collect on AM sales and the data we collect about the health of animals. The results showed that farmers who decrease their use of Category 1 AMs after the implementation of the regulation had no more mastitis cases [than farmers who did not decrease AMU] and there was no change in the number of cows sent to the slaughterhouse*.” (Veterinarian 4). Producers' perceptions were more heterogeneous. Some of them agreed on this topic: “*No joke, we changed our dry off AMs. It was a Category 1, we stopped using it. We're using another one, but it had no consequence on the herd*.” (Producer 8). However, many producers reported that there was a decrease in animal health and welfare: “*We have no tests, we don't have the AMs we need, so the cow loses a teat, she loses a quarter, and this is an economic loss. Sometimes you lose the cow and even if you don't lose the cow, the cow has lost a quarter*.” (Producer 20).

Veterinarians and producers explained that they made changes to their preventive practices. They all reported that the number of diagnostic tests performed each day increased and that they saw a shift toward more preventive medicine. Thus, one veterinarian explained: “*I have changed my practices. I focus more on prevention, and I do a lot more diagnostic testing*.” (Veterinarian 15). Other changes were reported such as better ventilation within the barn, increase in dry cow selective treatment, change in bedding, better genetics, change in feeding, use of internal teat sealants, increase in body temperature surveillance.

Knowledge of AMU and AMR and economic consequences were described as barriers to the implementation of the regulation, but these aspects were also reported as impacts by participants. Indeed, both producers and veterinarians had the perception that they had a better understanding of AMU and AMR since the implementation of the regulation. For instance, one veterinarian mentioned: “*We did a lot of awareness with farmers about preventive medicine, mastitis prevention, making sure that teat dip was done properly, and that the milking technique was perfect.”* (Veterinarian 6). Moreover, as veterinarians anticipated, the regulation did not seem to affect their practice economically. Indeed, one veterinarian mentioned: “*We sold less AMs, so it's certain that the turnover on drugs has decreased enormously, but the turnover in the laboratory has increased*.” (Veterinarian 11). Some producers who had some concerns about the risk of economic consequences finally perceived that there was no increase in cost per animal. Thus, one producer explained: “*I would say that, in the end, it costs us less because AMs are expensive and I used to use more of them, so in the end, I use less, so it costs me less*.” (Producer 14). However, other producers reported a decrease in economic benefit following the implementation of the regulation: “*Of course, if you treat with, for example, [ampicillin] or another product, it's 4 days of treatment, plus 2 days of milk withdrawal. So, there are definitely financial consequences attached to that.*..” (Producer 17).

## 4. Discussion

This study aimed to identify and describe the main barriers and facilitators encountered by dairy producers and dairy cattle veterinarians regarding the implementation of a regulation restricting the use, in food-producing animals only, of Category 1 AMs, in the province of Québec, Canada. The barriers and facilitators varied between veterinarians and producers.

One major barrier to the regulation implementation was the delays related to diagnostic tests. The text regarding the regulation does not provide clear guidance regarding the requirement for diagnostic testing before using a Category 1 AMs. It only states that it is necessary to justify that an AMs of lesser importance cannot be used before using a Category 1 AM. However, the use of diagnostic and susceptibility tests are one of the most relevant ways to justify veterinarians' choice regarding the use of a Category 1 AMs. Rapid diagnosis of disease is not always feasible in practice, due to the time lag between sampling and the reception of laboratory results. This challenge is exacerbated for producers who live in remote areas where the sample transit between the farm, the veterinary clinic and the laboratory is even more complicated due to a greater distance between these three infrastructures, and due to difficulties in accessing veterinary services in these regions. Delays in diagnosis have been described previously as a barrier to reduce AMU in several studies and in other countries (9, 13–15). This finding underlines the need to invest in diagnostic tests available in clinics, at the farm or to facilitate the direct sending by the producer to the diagnostic laboratory. This is in agreement with the results of Donadeu et al. (16), which emphasized the need for more diagnostic tools in dairies (16). What is encouraging is the arrival of bacterial identification using matrix-assisted laser desorption/ionization time-of-flight (MALDI-TOF) mass spectrometry in diagnostic laboratories which now allows for great speed in sending results, often within 24 h.

A second major barrier was the lack of access to alternatives to Category 1 AMs to prevent and treat some diseases. Indeed, in Canada, most of AMs treatments used for mastitis are intra-mammary infusions (17). Treatment duration ranged from 1 to 9 days and the only four commercial products available contained: 1) Pirlimycin (50 mg/10mL), 2) Ceftiofur (125 mg/10mL), 3) Cephapirin (200 mg/10mL), and 4), prior to 2019 and discontinued after, a combination of Penicillin G Procaine 100,000 I.U., Dihydrostreptomycin 100 mg, Novobiocin 150 mg, Polymyxin B Sulfate 50,000 I.U., Hydrocortisone Acetate 20 mg, Hydrocortisone Sodium Succinate 12.5 mg., per 10 mL (18). However, between June 2019 and May 2020, most of those AMs (e.g., for clinical mastitis and dry-cow treatments and all injectable tetracyclines) were not available or discontinued from supplies of veterinary medicine for different reasons such as commercial decisions and consequences of the COVID-19 pandemic except Category 1 AMs (JP. Roy, personal communication, January 30, 2021). Even if this situation may be exceptional and due to external factors, it directly affected the capability and opportunity drivers of behavior change for producers and veterinarians during and after this period. This barrier underlines the importance of supporting the accessibility to different treatment options before implementing similar regulations in the future.

Our findings suggest that attitudes toward the regulation varied between and across veterinarians and producers. The Kubler-Ross change curve explains individuals' reaction to change into five stages: denial, frustration, bargaining, depression and acceptance (19). The model suggests that an individual must move across those stages to accept change, which can be facilitated by information, communication, emotional support, and guidance. In our study, it seems that a small number of individuals were still in the frustration and bargaining phases. For example, a small number of producers and veterinarians argued that they did not have time to prepare and adjust their practice before the implementation of the regulation and believed that they were not adequately informed about the regulation and the possible alternatives. Some producers also felt that the legislation was unfair for them, as surrounding Canadian provinces haven't implemented similar regulation so far. This barrier underlines the importance of setting up an identification system or a label so that the milk and its added value can be identified by the consumer, at least on the Québec market.

In reality, education and information were offered to veterinarians before the implementation of the regulation, as they were required to attend a mandatory training course on AMR and AMU in April 2015. However, this training was not designed specifically to inform veterinarians and producers about the new regulation, but aimed to train them about better AMU practices in veterinary medicine (3). Veterinarians were also encouraged to offer optional training to producers of their regions, but not all producers from our study mentioned that their clinics proposed such training. All study participants had a good level of knowledge about AMU and AMR, but several reported problems with understanding how to apply the new regulation in specific contexts (e.g., when no alternatives are available on the market). This study supports the idea that education and information dissemination to veterinarians and producers are key to facilitate behavior changes and increase acceptability of regulations.

Veterinarians can play a central role in informing producers on farms as part of their regular practices. In our study, a trust relationship acted as a facilitator for most veterinarians and producers, most of whom explained that the veterinarian was a considerable support for them. Another study of communication and its impact on dairy herd health management in Sweden also found that a good veterinarian-client relationship and good communication skills were needed to initiate behavior change (20). Interestingly, some studies reported that veterinarians generally lack communication skills (21, 22). A Canadian study on biosecurity risk assessment in dairies also suggested that veterinarians do not have sufficient tools and skills to communicate their knowledge to producers (23). In a systematic review of the role of communication in veterinary clinical practice, Pun's research (24) also concluded that veterinarians do not have the communication skills to address complex problems that they are facing in the daily routine. Strengthening communication skills in the veterinary curriculum should be considered as part of the solution to changing on-farm practices.

While veterinarians did not mention any impact on animals, a small number of producers perceived that the implementation of the regulation was at the expense of animal health and welfare. This is coherent with findings from a study performed in the United Kingdom where large animal producers were reluctant to reduce AMU because it might affect animal health (25). We did not find any study demonstrating the negative consequences of AM reduction in the health and welfare of dairy cows during lactation. A systematic review conducted in 2010 on pork and broiler chicken production concluded that there was no clear evidence that reducing AMU could negatively impact the health of animals (26). Moreover, in the Netherlands, an overall reduction of the use of AMs by approximately 70% in 2015 compared to the index year 2009 has been attained, thereby looking at how to reduce further in the next year (27, 28). This reduction did not affect animal health or welfare (29).

Our results also revealed that even though producers were unclear about the actual economic consequences of the regulation, they feared economic losses. A study conducted in Canada on producers' willingness to reduce AMU in dairies concluded that a major barrier to reducing AMU was the investment in facilities and management practices (8). Scientific evidence on the economic impacts of reducing AMU is scarce and not consistent. Lhermie et al. (30) developed an economic model assessing the impact of restricting AMU in dairies. They found that policies aimed to reduce AMU would have minor economic effect on the industry. Evaluating the global cost of AM reduction is difficult, as when reducing AMU, producers generally increase prevention and biosecurity measures to protect animal health. Despite the cost of the implementation of such alternative measures to AMs, no or few negative economic consequences were detected in previous studies (31–34).

Producers and veterinarians who participated in our study perceived that there was an increase in preventive activities, such as vaccination or the use of teat sealants, in dairy farms after the implementation of the regulation. Numerous studies showed that an increase of management-related preventive activities such as vaccination, implementation of biosecurity measures, regular diagnostic testing and genetic selection, are essential to achieve a durable decrease in the use of AMs in animal production without negatively affecting animal health (9, 35, 36). It is not possible with this study design to confirm an increase in preventive activities, neither to attribute those possible changes to the new regulation. Programs promoting biosecurity and traceability exist in Canada, and a new program was implemented in dairy farms during the same period, namely the Canadian ProAction Initiative, a national quality assurance program developed by the dairy industry (37). ProAction consists of six modules (milk quality, food safety, animal care, traceability, environmental sustainability, and biosecurity), which promote national standards for on-farm practices. It is possible that producers have improved their management and preventive practices in order to meet these biosecurity standards. Synergies between preventive programs are desirable and should be encouraged. More research is needed to confirm changes in preventive practices and to attribute these changes to programs and/or regulations.

Unlike quantitative study design, the purpose of qualitative research is to characterize important aspects of people's perceptions, opinions, and beliefs about a subject, and not to measure their distribution in a population. Moreover, in our study, we used two different sampling strategies with the aim to gain a rich representation of producers and veterinarians from different regions and contexts. On the other hand, this sampling strategy may have favored the participation of individuals whose perspective does not reflect most producers and veterinarians, even though saturation was obtained. For example, producers and veterinarians who did not have issues with the implementation of the regulation could have shown a lack of interest in the study. Therefore, the generalization of the results to producers and veterinarians of Québec must be done with caution. Indeed, because the authors were already experienced with AMU in dairy farms, they could have been biased and have had misleading opinions about the regulation. This could happen when the researcher interprets the data to support his hypothesis or avoid data that does not support his hypothesis. To limit this bias, the codebook was developed with several collaborators (38). Moreover, our results show that the COM-B model can be applied successfully in the context of antimicrobial restrictions. Indeed, supporting our results on the COM-B model allowed us to better structure our analysis and, therefore, allowed us to gain a deep understanding of the current situation regarding the implementation of the regulation.

## 5. Conclusion

This study explored the barriers, facilitators, and perceived collateral impacts for dairy producers and veterinarians, of implementing a regulation aiming to restrict the use of Category 1 AMs in Québec, Canada. Major barriers included the lack of availability of alternative treatments to Category 1 AMs and long delays to obtain the results of diagnostic tests. Facilitators included education and access to training. These elements should be considered in the future before implementing similar regulations in order to maximize the acceptability of and compliance with this type of regulation. Finally, producers and veterinarians mentioned possible negative consequences on animal health and economic efficiency of their production, as well as an increase in preventive activities on farms. Further research investigating the effectiveness of regulations restricting AMU in animal production should integrate an evaluation of these impacts.

## Data availability statement

The raw data supporting the conclusions of this article will be made available by the authors, without undue reservation.

## Ethics statement

The studies involving human participants were reviewed and approved by the Université de Montréal's ethical board (Comité d'éthique de la recherche en sciences et en santé; certificate number CERSES-20-158-D). The patients/participants provided their written informed consent to participate in this study.

## Author contributions

NM, SD, CA, and J-PR contributed to the conception and design of the study. NM and CA analyzed the data and identified emerging themes. NM wrote the first draft of the manuscript. All authors contributed to manuscript revision, read, and approved the submitted version.
