# Corrigendum: Barriers and facilitators to implementing a new regulation restricting antimicrobial use in dairy production in Québec, Canada: a qualitative study

**DOI:** 10.3389/fvets.2024.1487705

**Published:** 2024-09-23

**Authors:** Nikky Millar, Simon Dufour, Hélène Lardé, Jean-Philippe Roy, Catherine Belloc, David Francoz, Marie-Ève Paradis, Marie Archambault, John Morris Fairbrother, Cécile Aenishaenslin

**Affiliations:** ^1^Department of Pathology and Microbiology, Faculty of Veterinary Medicine, Université de Montréal, Saint-Hyacinthe, QC, Canada; ^2^Groupe de recherche en épidémiologie des zoonoses et santé publique (GREZOSP), Faculty of Veterinary Medicine, Université de Montréal, Saint-Hyacinthe, QC, Canada; ^3^Centre de recherche en santé publique, Université de Montréal et Centre intégré de santé et de services sociaux du Québec du Centre-Sud-de-l'Île-de-Montréal, Montréal, QC, Canada; ^4^Fond de recherche Nature et technologies du Québec (FRQNT)—Regroupement FRQNT Op+lait, Saint-Hyacinthe, QC, Canada; ^5^Ross University School of Veterinary Medicine, Basseterre, Saint Kitts and Nevis; ^6^Department of Clinical Sciences, Faculty of Veterinary Medicine, Université de Montréal, Saint-Hyacinthe, QC, Canada; ^7^Oniris, INRAE, BIOEPAR, Nantes, France; ^8^Association des médecins vétérinaires praticiens du Québec, Saint-Hyacinthe, QC, Canada

**Keywords:** antimicrobials, antimicrobial resistance, legislation, animals, behavior, individual interview, qualitative research

In the published article, there was an error in Dr. Catherine Belloc's affiliation.

The affiliation “Biologie, Épidémiologie et Analyses de risque en santé animale (BIOEPAR), ONIRIS-INRAE, Nantes, France”, was assigned to Dr. Belloc; this should be replaced with “Oniris, INRAE, BIOEPAR, Nantes, France”.

The authors apologize for this error and state that this does not change the scientific conclusions of the article in any way. The original article has been updated.

