# Supplementary material for: Barriers and facilitators to implementing a new regulation restricting antimicrobial use in dairy production in Québec, Canada: A qualitative study
Source: Front Vet Sci. 2023 Mar 16;10:1025781. doi: 10.3389/fvets.2023.1025781 (PMC10060835; doi:10.3389/fvets.2023.1025781)
Supplement: Supplementary file 1 [file Data_Sheet_1.PDF]

Supplementary material 1

Interview guide for producers with open-ended questions regarding the implementation of the new regulation restricting the use of Category 1 antimicrobials

Introduction :

Let me introduce myself, I am X. I am a veterinarian and researcher at the Faculty of Veterinary Medicine at the University of Montreal. With five other researchers (Cécile Aenishaenslin, Simon Dufour, Jean-Philippe Roy, Hélène Lardé and Christine Fourichon), we are working on a project about the impact of a new regulation restricting the use of high importance antibiotics (Category 1 antimicrobials) in dairies and on herd health management. Indeed, as you know, since February 25, 2019, the use of Category 1 antibiotics for preventive purposes is prohibited and their use for curative purposes is limited *(Loi sur la protection sanitaire des animaux (chapter P-42, a. 55.9, 1st para., par. 7° and 11°). Are you aware of the new regulation? Would you like a reminder?

During this interview, which will last about 30-35 minutes, I propose to explore several themes, such as your knowledge about antibiotics and antibiotic resistance, what leads you to administer an antibiotic to an animal and the impacts of this new regulation on your practices.

This study is being conducted as part of a PhD's thesis. Our interview is confidential. Only anonymous extracts, not allowing you to be identified, will be quoted in the study. (Here, explain the anonymity and ask the participant to read and sign the consent form.)

- *Do you have any questions before we begin?*

***Animal Health Protection Act** (chapter P-42, s. 55.9).

''***Bylaw 1.1.*** *Administration, for curative use, of a medication belonging to the category of very high importance antimicrobials to an animal that will be used (or from which products will be used) as human food is restricted to clinical cases that are not treatable with an antimicrobial of a lower importance category based on, for example, a culture and susceptibility testing.*

Bylaw 1.2. *It is forbidden to administer for a preventive purpose a medication that belongs to the category of very high importance antimicrobials to an animal that will be used (or from which products will be used) as human food* ''

Interview guide – Producers –

Theme 1 : Introduction [3 min]

QUESTION 1: *Can you please state your name and tell me about your farm briefly?*

Theme 2: Perception of the regulation before its implementation [10 min]

- QUESTION 2: *What was your opinion about this new regulation before it came into effect? [Reference to the COM-B model: capability (Psychological) - knowledge, beliefs]*

Sub-Questions

- *How did you learn about the purpose of this regulation and about reducing antibiotic use in general? [Reference to the COM-B model: motivation - knowledge, social influences]*

- Why do you think this regulation should be put in place? [Reference to the COM-B model: motivation: attitude]

- What consequences do you think this regulation would have on your production? [Reference to the COM-B model: motivation - beliefs about consequences]

Theme 3: Barriers and facilitators to the implementation of the new regulation [15 min]

- QUESTION 3: *How did you experience the implementation of this new regulation? [Reference to the COM-B model: capability (Psychological) - Emotions]*

Sub-Questions

- *What are the elements/factors that encouraged changes on your farm during the implementation of this regulation?*
- How difficult or easy is it for you to adopt the requirements of this new regulation (farming context, beliefs, interests)? [Reference to the COM-B model: motivation - beliefs about capabilities, intention, goals] [Reference to the COM-B model: capability [Psychological - Behavioural regulation]
- Were there any barriers to reducing the use of Class 1 antibiotics? [Reference to the COM-B model:Physical opportunity - environment context and resources]
- Can you tell me about the positive and negative impacts of this regulation? [Reference to the COM-B model: Reflective motivation -beliefs about consequences]
- Can you tell me about the role of the veterinarian in the implementation of this regulation? [Reference to the COM-B model: motivation-social influence] What was your veterinarian's role in this transition?
- What has changed for you since the implementation of the new regulation?
- How have your practices changed? [Reference to the COM-B model: reflective motivation - Emotions]

Theme 4: Openness [5 min]

QUESTION 4: *How do you see the future of the dairy industry in the face of new measures to restrict the use of antibiotics?*

Sub-questions

- What do you think are the ideal strategies for reducing antibiotic use?
- What would have made it easier for dairy producers to implement these regulations?
- What do you think are the public's perceptions and concerns about antibiotic use? Could it change with this kind of regulation?
- Québec is the only province in North America that has implemented this type of regulation, do you think it may have had consequences/ may have consequences?

Supplementary material 1

Interview guide for dairy veterinarians with open-ended questions regarding the implementation of the new regulation restricting the use of Category 1 antimicrobials

Introduction :

Let me introduce myself, I am X. I am a veterinarian and researcher at the Faculty of Veterinary Medicine at the University of Montreal. With five other researchers (Cécile Aenishaenslin, Simon Dufour, Jean-Philippe Roy, Hélène Lardé and Christine Fourichon), we are evaluating the impact of a new regulation on the use of high importance antibiotics (category 1) in cattle production and on herd health management. Indeed, as you know, since February 25, 2019, the use of Category 1 antibiotics for preventive purposes is prohibited and their use for curative purposes is limited *(Loi sur la protection sanitaire des animaux (chapitre P-42, a. 55.9, 1er al., par. 7° et 11°). During this interview, which will last approximately 30-35 minutes, I propose to explore several themes, such as your knowledge about antibiotics and antibiotic resistance, what leads you to prescribe/give an antibiotic to an animal, the impacts of this new regulation and your perception about the future of dairy production and the new measures.

This study is being conducted as part of a PhD's thesis. Our interview is confidential. Only anonymous extracts, not allowing you to be identified, will be quoted in the study. (Here, explain the anonymity and ask the participant to read and sign the consent form.)

- Do you have any questions before we begin?

***Animal Health Protection Act** (chapter P-42, s. 55.9).

''***Bylaw 1.1.*** *Administration, for curative use, of a medication belonging to the category of very high importance antimicrobials to an animal that will be used (or from which products will be used) as human food is restricted to clinical cases that are not treatable with an antimicrobial of a lower importance category based on, for example, a culture and susceptibility testing.*

Bylaw 1.2. *It is forbidden to administer for a preventive purpose a medication that belongs to the category of very high importance antimicrobials to an animal that will be used (or from which products will be used) as human food ''*

Interview guide – Veterinarians –

Theme 1: Introduction [5 min]

-QUESTION 1: *Can you please tell me your name and talk briefly about your practice?*

Theme 2: Perception of the regulation before its implementation [10 min]

- QUESTION 2: *What was your opinion of this new regulation before it came into effect? [Reference to the COM-B model: capability (Psychological) - knowledge, beliefs]*

Sub-Questions

- How did you learn about the purpose of this regulation and about reducing antibiotic use in general? [Reference to the COM-B model: motivation - knowledge, social influences]
- How important do you think it was to put such a regulation in place? [Reference to the COM-B model: motivation attitude]
- What consequences do you think this regulation could have had on your practice? [Reference to the COM-B model: motivation - beliefs about consequences]
- What was your role in making producers aware of this regulation?
- How do you think this regulation was perceived by producers?

Theme 3: Barriers and facilitators to the implementation of the new regulation [15 min]

- QUESTION 3: *How have you experienced the changes caused by this new regulation? [Reference to the COM-B model: capability (Psychological) - Emotions]*

Sub-Questions

- How difficult or easy is it for you to adopt the requirements of this new regulation (breeding context, beliefs, interests)? [Reference to the COM-B model: motivation - beliefs about capabilities, intention, goals] [Reference to the COM-B model: capability (Psychological - Behavioural regulation]
- Were there any barriers to reducing the use of Class 1 antibiotics? [Reference to the COM-B model: physical opportunity - environment context and resources]
- Can you tell me about the benefits and consequences of the implementation of this regulation? [Reference to the COM-B model: reflective motivation - beliefs about consequences]
- Can you tell me about your role in the implementation of this regulation? [Reference to the COM-B model: motivation-social influence]
- Did you change some aspects of your antimicrobial use practices after the implementation of the regulation?
- Has your perception of this regulation changed since it came into effect? [Reference to the COM-B model: reflective motivation - Emotions]
- How did your clinic adapt its practices after the implementation of the regulation (or not)?

Theme 4: Openness [5 min]

- QUESTION 4: *How do you see the future of the dairy industry in the face of new measures to restrict antibiotic use?*

Sub-questions

- What do you see as the levers for action to reduce the use of antibiotics?
- What do you think are the public's perceptions and concerns about the use of antibiotics? Could it change with this kind of regulation?
- Québec is the only province in North America that has implemented this type of regulation, do you think it may have had consequences/ may have consequences?
