# Supplementary material for: Barriers and facilitators to implementing a new regulation restricting antimicrobial use in dairy production in Québec, Canada: A qualitative study
Source: Front Vet Sci. 2023 Mar 16;10:1025781. doi: 10.3389/fvets.2023.1025781 (PMC10060835; doi:10.3389/fvets.2023.1025781)
Supplement: Supplementary file 2 [file Data_Sheet_2.PDF]

Supplementary material 2

Codebook based on the COM-B model developed using an inductive-deductive approach after the analysis of 27 verbatim of dairy producers and 15 verbatim of dairy cattle veterinarians taking part in individual interviews about barriers and facilitators of the implementation of a regulation restricting the use of Very High importance antimicrobial for human medicine in animal production in the province of Québec, Canada.

Code	***Definition***
Preparation for the implementation of the regulation	Any reference to how producers and veterinarians prepared themselves to the implementation of the regulation regarding antimicrobial use.
Time between the announcement of the regulation and the implementation of the regulation (preparation time)	Refer to the amount of time producers and veterinarians have had to prepare for the implementation of the regulation.
Access to training and education about the regulation, antimicrobial use or antimicrobial resistance before the implementation of the regulation (access to training and education)	Refer to the possibility (or the absence of a possibility) for veterinarians/producers to participate in any training/education program about the new regulation, antimicrobial resistance, or antimicrobial use before the implementation of the regulation.
How producers/veterinarians were informed about the regulation (information process)	Refer to how producers/veterinarians were informed about the implementation of the regulation.<br>Ex: Veterinarian, Newspapers
Antimicrobial stockpile before the implementation of the regulation	Refer to the action of making antimicrobial stockpile before the implementation of the regulation.
Motivation to change antimicrobial use	Any reference to producers' or veterinarians' motivation (or reluctance) to change their practices due to the implementation of the regulation.
Attitudes (Opinion) of the veterinarians and producers about the regulation (Motivation - automatic)	Refer to veterinarians' and producers' attitude (opinion about the regulation restricting Category 1 antimicrobial use before its implementation.
Protecting public health (Motivation – reflective)	Refer to the veterinarians' or producers' beliefs about the protection of public health as a motivation to change (or not to change) antimicrobial use.

Protecting producers' animal health (Motivation – reflective)	Refer to the veterinarians' or producers' beliefs about the protection of animal health as a motivation to change (or not to change) antimicrobial use.<br>e.g.: The regulation will decrease the wellbeing of animals; the regulation is good for animals.
Protecting the health of their family and employees (Motivation – reflective)	Refer to the veterinarians' or producers' beliefs about the protection of the family as a motivation to change (or not to change) antimicrobial use.<br>e.g.: Use of antimicrobials on the farms as a direct impact on my family
Concerned about disciplinary actions (Motivation – automatic)	Refer to the veterinarians' or producers' concern about disciplinary action as a motivation to change (or not to change) antimicrobial use.
Other	Refer to the veterinarians' or producers' motivation to change antimicrobial use that has not been categorized before.
Capability to change antimicrobial use	Any reference to the capability as a factor (or as an unimportant factor) to change antimicrobial use.
Knowledge about antimicrobial use and antimicrobial resistance (Capability – psychological)	Refer to the knowledge of producers and veterinarians about the regulation, about Category 1 antimicrobial alternatives and diagnostic tests as an element of change (or absence of change) to antimicrobial use.
Difficulty to manipulate cows (Capability - physical)	Refer to the difficulty to manipulate cows when producers must use alternative treatments to Category 1.
Knowledge about how to enforce the regulation (capability - psychological)	Refer to veterinarians' knowledge about how easy (or difficult) it is to enforce the regulation.
Antimicrobial use profile as a capacity to change antimicrobial use (Capability - psychological)	Refer to the producers' and veterinarians' subjective opinion on how much antimicrobials they were using before the implementation of the regulation
Other	Refer to the veterinarians' or producers' capability to change antimicrobial use that has not been categorized before.
Opportunity to change antimicrobial use	Any reference to physical opportunity as a factor (or as an unimportant factor) to change antimicrobial use.

Availability of alternatives to Category 1 antimicrobials (Opportunity - physical)	Refer to the availability of alternatives to Category 1 antimicrobials as a factor to change (or not to change) antimicrobial use.
Access to diagnostic tests (Opportunity- physical)	Refer to the presence (or absence) of diagnostic tests as a factor to change (or not to change) antimicrobial use.
Proximity to a veterinary facility (Opportunity - physical)	Refer to the proximity to a veterinary facility as a factor to change (or not to change) antimicrobial use.
Economic reasons (Opportunity - physical)	Refer to the price of an alternative to Category 1 antimicrobials as a factor to change (or not to change) antimicrobial use.<br>e.g.: price of diagnostic tests, price of veterinary consultation, price of vaccines
Time before treating an animal (Opportunity - physical)	Refer to the amount of time between the moment a cow felt sick and the moment it is possible to cure it as a factor to change (or not to change) antimicrobial use.<br>e.g.: time to have results from diagnostic tests, time for the veterinarian to come to the farm
Peer influence on changing AMU (Opportunity - Social)	Refer to the influence of peers (veterinarians or producers) as a factor to change (or not to change) antimicrobial use.<br>e.g.: explanation by the veterinarian, colleagues who want to change antimicrobial use on the farm, pressure to give Category 1 antimicrobials without diagnostic tests.
Competition with peers (Opportunity - social)	Refer to the comparison between peers as a factor to change (or not to change) antimicrobial use.<br>e.g.: comparison with other provinces, colleagues
Other	Refer to the veterinarians' or producers' opportunities to change antimicrobial use that has not been categorized before.
Consequences/Impact of the regulation	Any reference to the consequences of the new regulation.
Impact – Improvement antimicrobial use	Refer to any change (or absence of change) in the type of antimicrobials that are used and the amount of antimicrobials that are used.

Impact – Knowledge about AMU, AMR	Refer to any change (or absence of change) in the knowledge of producers or veterinarians about antimicrobial use and antimicrobial resistance.
Impact – Animal Health and Wellbeing	Refer to any animal health or animal wellbeing change (or absence of change) following the implementation of the regulation.<br>e.g.: mastitis, milk quality
Impact – Direct or indirect Economic consequences	Refer to the economic consequences associated with the implementation of the new regulation.
Impact – Change in preventive and management practices	Refer to any change (or absence of change) in preventive and management practices.<br>e.g.: vaccines, change in routine, in treatment procedure, working schedule, diagnostic tests
Impact - Image of the dairy industry	Refer to change (or absence of change) of their perception towards dairy production after the implementation of the regulation.
Illegal practice	Refer to producers' and veterinarians' illegal practice after the implementation of the regulation.<br>e.g.: illegal antimicrobial use
Other	Refer to any other impact of the implementation of the regulation.
Future of the dairy industry	Any reference to the future of the dairy industry regarding antimicrobial use.
Additional regulation to restrict antimicrobial use	Refer to the implementation of new restrictions in order to reduce antimicrobial use in the future.
Measures to improve management practices	Refer to the implementation of new management practices on farms in order to reduce antimicrobial use.<br>e.g.: changing for better litter, changing alimentation
Research and development of drugs	Refer to the importance of research and the development of new vaccines, antimicrobials, and order medicine in order to be able to reduce antimicrobial use without compromising animal health.
Education and communication tools for veterinarians and producers	Refer to the increase of education program and communication tools for veterinarians and producers in order to reduce antimicrobial use.

	e.g.: optional training for producers
Raising public awareness	Refer to the importance of raising public awareness in order to reduce antimicrobial use.
Benchmarking program	Refer to the implementation of a benchmarking program between producers/veterinarians in order to reduce antimicrobial use in the future.
Attitude about the future	Refer to the producers' or veterinarians' attitude about reducing antimicrobial use in the future.
Animal wellbeing regarding antimicrobial use reduction in the future	Refer to the animal wellbeing because of antimicrobial use reduction in the future.
Overcoming barriers to unfair competition	Refer to peer competition as an element that enhances the reluctance to reduce antimicrobial use in the future.
Other	Any reference to the future of the dairy industry that has not been listed above.
